# Enhanced CO_2_ Adsorption on Activated Carbon Fibers Grafted with Nitrogen-Doped Carbon Nanotubes

**DOI:** 10.3390/ma10050511

**Published:** 2017-05-07

**Authors:** Yu-Chun Chiang, Wei-Lien Hsu, Shih-Yu Lin, Ruey-Shin Juang

**Affiliations:** 1 Department of Mechanical Engineering, Yuan Ze University, Chung-Li, Taoyuan 32003, Taiwan; ycchiang@saturn.yzu.edu.tw (Y.-C.C.); s1005906@mail.yzu.edu.tw (W.-L.H.); 2 Fuel Cell Center, Yuan Ze University, Chung-Li, Taoyuan 32003, Taiwan; 3 Department of Mechanical Engineering, Hwa Hsia Institute of Technology, Chung-Ho, New Taipei City 235, Taiwan; jiun@cc.hwh.edu.tw; 4 Department of Chemical and Materials Engineering, Chang Gung University, Guishan, Taoyuan 33302, Taiwan; 5 Division of Nephrology, Department of Internal Medicine, Chang Gung Memorial Hospital, Linkou 33305, Taiwan; 6 Department of Safety, Health and Environmental Engineering, Ming Chi University of Technology, Taishan, New Taipei City 24301, Taiwan

**Keywords:** activated carbon fibers, carbon nanotubes, composites, carbon dioxide, adsorption

## Abstract

In this paper, multiscale composites formed by grafting N-doped carbon nanotubes (CNs) on the surface of polyamide (PAN)-based activated carbon fibers (ACFs) were investigated and their adsorption performance for CO_2_ was determined. The spaghetti-like and randomly oriented CNs were homogeneously grown onto ACFs. The pre-immersion of cobalt(II) ions for ACFs made the CNs grow above with a large pore size distribution, decreased the oxidation resistance, and exhibited different predominant N-functionalities after chemical vapor deposition processes. Specifically, the CNs grafted on ACFs with or without pre-immersion of cobalt(II) ions were characterized by the pyridine-like structures of six-member rings or pyrrolic/amine moieties, respectively. In addition, the loss of microporosity on the specific surface area and pore volume exceeded the gain from the generation of the defects from CNs. The adsorption capacity of CO_2_ decreased gradually with increasing temperature, implying that CO_2_ adsorption was exothermic. The adsorption capacities of CO_2_ at 25 °C and 1 atm were between 1.53 and 1.92 mmol/g and the Freundlich equation fit the adsorption data well. The isosteric enthalpy of adsorption, implying physical adsorption, indicated that the growth of CNTs on the ACFs benefit CO_2_ adsorption.

## 1. Introduction

Global warming is one of the most important recent environmental concerns, which is mostly caused by anthropogenic activities. Carbon dioxide (CO_2_) is believed to be the primary greenhouse gas emitted from human activities, accounting for about 96% of greenhouse gases from human activities in Taiwan [1] and 84% in the USA [2]. CO_2_ capture and sequestration (CCS) is a set of technologies that can greatly reduce CO_2_ emissions from new and existing emission sources and is considered as an effective approach in mitigating global warming. Since most anthropogenic CO_2_ was a byproduct of the combustion of fossil fuels from coal-fired plants and large industrial sources, CO_2_ capture technologies were commonly classified as pre-combustion capture, post-combustion capture, and oxy-combustion systems [3]. Among the different CCS methods available, post-combustion capture appears to be the most feasible approach as it can be retrofitted to existing power plants without upgrading or modifying the existing systems [4]. Accordingly, much effort has been expended to develop various physical and chemical methods for post-combustion CO_2_ capture [5].

The capture technology is the most important for cost-effective removal of CO_2_ from the atmosphere since the expenses during capture processes account for about 70% of the overall costs in CCS [6]. Various CO_2_ capture technologies, such as absorption, adsorption, cryogenics, and membranes, have been widely studied. Among them, absorption-regeneration technologies have been recognized as the most effective methods, where the processes by using ammonia-based or amine-based absorbents have received much attention [7]. However, the comparatively high energy demand of absorption processes restricts their applications. In 2005, the Intergovernmental Panel on Climate Change (IPCC) special report disclosed adsorption methods might be promising processes to separate CO_2_ from gases by choosing appropriate adsorbents [8]. In this respect, several possible adsorbents, including activated carbon [9], activated carbon fibers (ACFs) [6], zeolite [10], silica adsorbents [11], or carbon nanotubes (CNTs) [12], have been considered as potential candidates for CO_2_ capture.

The specific adsorption capacity may be enhanced by increasing the affinity of the adsorbent surface to CO_2_. Due to the acidic role of CO_2_ (a weak Lewis acid), it is expected that the introduction of Lewis bases onto the activated carbon surfaces may favor CO_2_ capture performance of these materials. Nitrogen enrichment was reported to be effective in introducing basic functionalities that enhanced the specific adsorbent-adsorbate interaction for CO_2_ [13]. The N functional groups on the surface of adsorbents gave the Lewis-base active sites to the materials, inducing electron-donor properties, which might be relevant for the capture of acidic gases, such as CO_2_ [14]. The methods of obtaining N-enriched carbons included the introduction of N in precursors of carbons, the use of activation agents containing N, and the exposure to nitrogen compounds at elevated temperatures [15]. 

Among of the known adsorbents, ACFs are featured with the advantages of small fiber diameter, large specific surface area, uniform pore size distribution, and rapid adsorption/desorption rate. In addition, the hierarchical composites composed of microscaled substrates decorated with nanoscaled materials have attracted much attention due to several synergistic effects being realized from multidisciplinary researchers [16]. The ideas of growing carbon nanotubes (CNTs) onto the carbon fibers (CFs) could be traced back to the studies of Downs and Baker [17,18]. Direct growth of CNTs on the CF surfaces has been proposed to modify the interface of the fibers and tailor the physical properties [19]. Grafting CNTs on the surface of carbon fibers is an effective method to create mechanical interlocking, and/or local stiffening, at the fiber/matrix interface, all of which may improve stress transfer and interfacial properties [16,19]. The growth of CNTs on the surface of fibers along the radial orientation could also increase the transverse reinforcement [20]. The interfacial shear strength of the CFs is also shown to be significantly improved due to the CNTs grown on the CF surface [21]. Greef et al. [22] have compared two processes of growing CNTs directly onto the CFs and found that the growth temperature was the most critical parameter. They indicated that 700 °C was the optimal temperature to obtain a high number of CNTs grafted homogeneously on the CFs.

The catalytic growth of CNTs strongly depends on the process conditions, such as carbon sources and their feeding rates, types, and concentrations of the catalysts, temperature, and atmosphere. When the CNTs were doped with N atoms through a replacement of C atoms, the local physical properties around N atoms generated a significant change, resulting in a change of local chemical reactivity. This change increased the binding energy and the possibility of gas molecules interacting with N-doped CNTs (CNs). The adsorption capacity of CO_2_ on KOH-modified ACFs was achieved to 250 mg/g at 25 °C and 1 atm, while that on as-received ACFs was only 140 mg/g [6]. The adsorption amount of CO_2_ on CNTs and CNTs modified by 3-aminopropyltriethoxysilane decreased with temperature, but increased with water content in air at 0–7% [12].

The growth of CNTs directly on the CF surface has gained much attention for the applications of CF-reinforced polymer composites. The growth of CNTs has been considered as one of surface modification methods of CFs to improve interface shear strength between CFs and the matrix. However, the studies related to the characteristics and applications of the CNTs grown on the ACFs are scarce in the literature. Tzeng et al. [23] have tried to utilize ACFs as the substrate to grow carbon nanofibers (CNFs), taking advantage of their large amounts of micropores to obtain a uniform dispersion of the nanosized catalytic metal ions for CNF growth. Kong et al. [24] fabricated a micro-nano-scaled CNT/hollow ACF composite and attempted to propose the possible formation mechanisms. However, these studies did not show the applications of their multi-scaled composites. Moreover, the publications about multi-scaled composites have seldom discussed the applications of CNs. Therefore, the objective of this paper is to investigate the physicochemical properties of ACFs grafted with CNs (CNs/ACFs) and determine their adsorption capacity of CO_2_.

## 2. Experimental Methods

### 2.1. Synthesis of CNs-Grafted ACFs

One commercial PAN-based ACF sample manufactured by Taiwan Carbon Technology Co. (Taichung, Taiwan) was used as the starting material (denoted as ACF). The chemical vapor deposition (CVD) method was used for the growth of CNs directly onto ACFs through an iron-catalyzed reaction in a tubular furnace system. The CVD system consisted of a horizontal tubular furnace with a three-zone heating and a quartz tube with a diameter of 2.54 cm. Acetonitrile was used as both the C and N sources and the ferrocene as the catalyst precursor, where the mixture of acetonitrile and ferrocene were prepared with a ratio of Fe/C = 1.3 (*w*/*w*). The ACF samples (9 cm × 8 cm, about 1.0 g) were settled inside the quartz tube and the mixture of acetonitrile and ferrocene was injected into the tubular furnace with at a rate of 1.2 mL/h using a syringe pump (KD Scientific). The process was carried out for 2 h at 900 °C in a flowing 5% H_2_/Ar gas mixture (500 sccm). To compare the effects of the catalyst compositions, the ACF samples were immersed in 0.05 M cobalt(II) acetate (Co(C_2_H_3_O_2_)_2_) solution and mixed for 3 h. Then the samples were dried at 100 °C for 24 h before being placed in the furnace. The products were denoted as CN1/ACF or CN2/ACF, depending on whether the ACF samples were without or with pre-immersion of the 0.05 M cobalt(II) acetate solution.

### 2.2. Characterization Techniques

The as-received ACF and CNs-grafted ACF were characterized using several techniques. The surface morphology of the samples was inspected using field emission scanning electron microscopy (FESEM, S-4700, Hitachi, Krefeld, Germany). The oxidation resistance of the samples was determined in flowing air (60 cm^3^/min) with a heating rate of 10 °C/min, using a thermogravimetric analyzer (Q500, TA Instrument, New Castle, DE, USA) to measure any changes in the weight of the sample as a function of temperature (TGA plot) and the rate of weight loss versus temperature (differential thermogravimetry, DTG, plot). X-ray photoelectron spectroscopy (XPS) was used to determine the number and type of functional groups present on the surface of the samples. The XPS spectra of all samples were obtained using a spectrophotometer (PHI 1600 ESCA, Perkin-Elmer, Waltham, MA, USA). A twin anode Mg X-ray source (hν = 1253.6 eV) was used, at a voltage of 15 kV and a power of 400 W. For calibration purposes, the C_1s_ electron binding energy that corresponds to graphitic carbon was set at 284.6 eV. A nonlinear least squares curve-fitting program (XPSPEAK software, version 4.1, The Chinese University of Hong Kong, Hong Kong, China) was used for deconvolution of the XPS spectra. The surface feature of the samples was probed by N_2_ adsorption/desorption isotherms measured at –196 °C using an ASAP 2020 (Micromeritics, Norcross, GA, USA) accelerated surface area and porosimetry system.

### 2.3. CO_2_ Adsorption

The CO_2_ adsorption isotherms on all samples were measured using a Micromeritics ASAP 2020 accelerated surface and porosimetry analysis system at 25 °C, 40 °C, or 55 °C. The sample (~0.08 g) was outgassed at 350 °C overnight to remove adsorbed contaminants prior to the measurement. The equilibration interval for each pressure point was 30 s. The temperature during the CO_2_ adsorption process was maintained by a circulating water bath thermostat. In this study, three common adsorption isotherms, including the Freundlich equation, Langmuir equation, and Toth equation, were used to fit the experimental data [25]. The Freundlich isotherm (Equation (1)) is an empirical equation that assumes heterogeneous adsorption due to the diversity of adsorption sites:

(1) $$q_{e}=K_{F}P^{1/n}$$

where *q_e_* (mmol/g, STP) is the equilibrium adsorption capacity, *P* (kPa) is the equilibrium gas pressure, *K_F_* [(mmol/g)(1/kPa)^1/n^] is the Freundlich adsorption coefficient, and *n* is a constant indicating the isotherm curvature. The parameter *n* is usually greater than unity, and the larger this value is, the more nonlinear the adsorption isotherm becomes. The parameters *n* and *K_F_* both usually decrease with increasing temperature.

The Langmuir isotherm assumes that the adsorption involves the attachment of only one layer of molecules to the surface, i.e., monolayer adsorption, which is shown in Equation (2):

(2) $$q_{e}=\frac{q_{m}K_{L}P}{1+K_{L}P}$$

where *q_m_* (mmol/g) is the monolayer adsorption capacity and *K_L_* (1/kPa) is the Langmuir adsorption equilibrium coefficient. Equation (3) shows the Toth isotherm, a three-parameter model that has been particularly used to describe gas-phase adsorption data. It reduces to the Henry type at low pressures and approaches the saturation limit at high pressures:

(3) $$q_{e}=\frac{q_{T}K_{T}P}{{\left[1+{\left(K_{T}P\right)}^{\;t}\right]}^{\;1/t}}$$

where *q_T_* (mmol/g) is the saturation adsorption capacity, *K_T_* (1/kPa) is the Toth adsorption equilibrium coefficient, and *t* is the degree of the heterogeneity of the adsorbent. When *t* equals unity, the above equation is identical to the Langmuir equation. 

Finally, the Clausius–Clapeyron equation, Equation (4), was used to calculate the isosteric enthalpy of adsorption:

(4) $$\ln P=A-\frac{Q_{st}}{R}\frac{1}{T}$$

where *Q*_st_ (kJ/mol) is the isosteric enthalpy of adsorption, *R* (8.314 J/mol/K) is the gas constant, *T* (K) is the adsorption temperature, and *A* is the coefficient.

## 3. Results and Discussion

### 3.1. Characterization of Various ACF Samples

**Figure 1:**
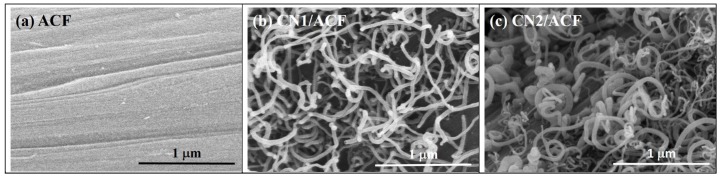
FESEM images of the samples: (**a**) as-received activated carbon fibers (ACF); (**b**) ACF grafted with N-doped carbon nanotubes (CN1/ACF); (**c**) ACF pre-immersed with cobalt(II) acetate and grafted with N-doped carbon nanotubes (CN2/ACF).

The FESEM images of the samples are shown in Figure 1. The diameter of a single fiber, as-received, was approximately 6.8 μm and the surface was smooth with typically well-defined striations almost parallel to the fiber axis (Figure 1a). After the conventional CVD process, the spaghetti-like and a slightly randomly oriented N-doped CNTs with a high density and homogeneity were grown onto the ACFs. The nanotubes on CN1/ACF were long with smaller and uniform diameters, and an average diameter of CN1 of about 30–60 nm. On the other hand, the nanotubes on CN2/ACF had a larger range of diameters with a mean diameter of CN2 of about 25–83 nm. Moreover, most catalyst particles were preferentially present at the end of the CNs, as observed in Figure 1b,c, indicating low chemical interactions between catalyst particles and the carbon fiber surface, hence, initiating the tip-growth mode. Some nanotubes had an open end, which is probably due to the falling-off of the catalyst particles, similar to the study of Greef et al. [22].

Kim et al. [21,26] reported that the catalyst coating and nanoparticle formation degraded the CF surface by inducing amorphous carbons and damaging graphitic layers. However, those defects were healed by the injected carbons and interfaced CNTs during the CVD process. Due to the difference in catalytic efficiency, the catalyst composition has a strong influence on CNT growth. Greef et al. [22] have found that bimetallic catalysts were more efficient and caused less surface damage than mono-metallic ones. The use of a bimetallic catalyst even led to the growth of CNTs at low temperatures to avoid the diffusion of the catalyst particles into CFs [27]. However, the results in this study exhibited that pre-immersion of cobalt(II) acetate solution for ACFs caused the growth of CNs with a less uniform diameter and an inferior degree of graphitization.

**Figure 2:**
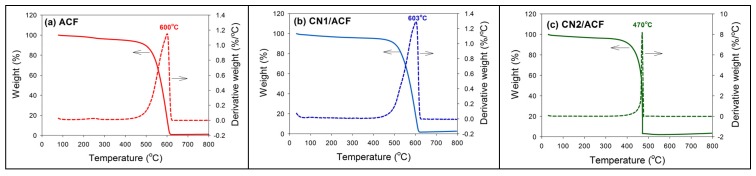
Thermogravimetric analysis (TGA) and differential thermogravimetry (DTG) profiles of the samples: (**a**) as-received activated carbon fibers (ACF); (**b**) ACF grafted with N-doped carbon nanotubes (CN1/ACF); (**c**) ACF pre-immersed with cobalt(II) acetate and grafted with N-doped carbon nanotubes (CN2/ACF).

The TGA profiles are shown in Figure 2. The temperatures for the maximum rate of weight loss (or oxidation) for ACF, CN1/ACF, and CN2/ACF were 600, 603, and 470 °C. Compared with Figure 2a,b, the oxidation resistance did not significantly change after the CVD process. It should be noted that the oxidation resistance decreased as the ACFs were pre-immersed in cobalt(II) acetate solution (Figure 2c). This could be attributed to the fact that Co(II) ions penetrated the interior of the ACFs during the CN growth at high temperatures, thus inducing amorphous carbons and damaging graphitic layers [21,26]. This phenomenon accelerated the oxidation of ACFs, the destruction of the structural and mechanical strength of ACFs, and was followed by a decrease in temperature for the maximum rate of weight loss. 

**Figure 3:**
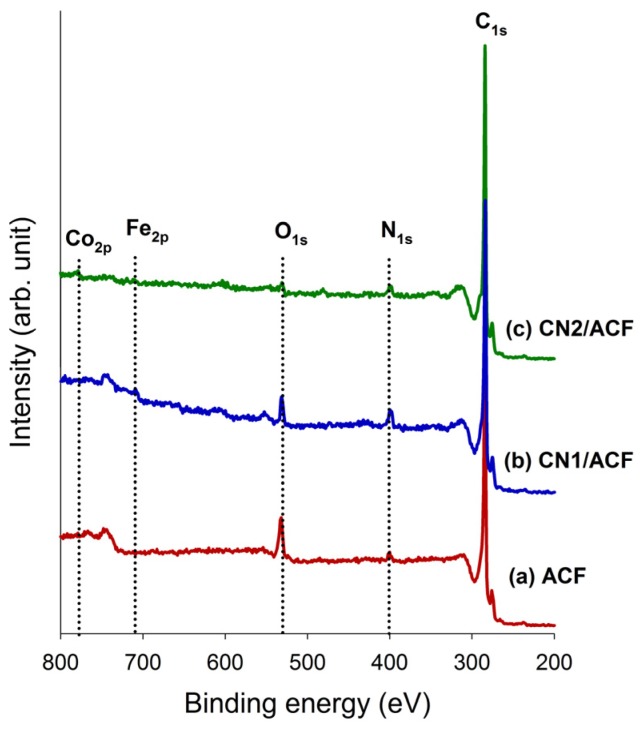
The X-ray photoelectron spectroscopy (XPS) survey scan spectra of the samples: (**a**) as-received activated carbon fibers (ACF); (**b**) ACF grafted with N-doped carbon nanotubes (CN1/ACF); (**c**) ACF pre-immersed with cobalt(II) acetate and grafted with N-doped carbon nanotubes (CN2/ACF).

The XPS survey scan spectra of the samples, shown in Figure 3, revealed that the major peaks in the scan spectra were due to the C_1s_, O_1s_, and N_1s_ photoelectrons. The surface atomic contents and ratios are summarized in Table 1. The as-received PAN-based ACFs contained about 2.43 at% of N_1s_ and 8.20 at% of O_1s_. The graft CNs on ACFs significantly increased the surface N_1s_ content, which could be attributed to the contribution from N atoms on CNs. Moreover, CN grafting resulted in a large decrease in O_1s_ content. This implied the Lewis-base active sites on the materials increased and electron-donor properties were predominant [14]. The ratios of N/C and O/C also reported a similar tendency. Small amounts of iron(III) or cobalt(II) were measured on CN1/ACF and CN2/ACF (Figure 3 and Table 1), which was attributed to the residues of catalysts for the growth of CN1 or CN2. The pre-immersion of cobalt(II) acetate solution for ACFs was expected to make the nitrogen atoms on the ACF surface drain off, or reduce the N_1s_ content due to the catalytic reactions of the cobalt(II). Under the circumstance of a single catalyst, the residue of iron(III) on the surface was about 0.69 at%. When the ACF was pre-immersed with cobalt(II) acetate, a smaller amount of iron(III) settling on the surface was observed and the atomic ratio of Co(II) to Fe(III) was about 4.7.

Furthermore, the predominant, discriminative, N functional groups were on CN1/ACF and CN2/ACF. Table 2 shows the results of curve-fitting for the high-resolution XPS N_1s_ spectra for all samples. According to the existing literature data, the N_1s_ spectra were decomposed into, at most, seven identified components. As seen from Table 2, the pyrrolic or amine moieties were the major groups on CN1/ACF, while the pyridine-type N was the main N-functionality on CN2/ACF. The patterns of N-functionality of the latter was much similar to that of as-received ACFs. To sum up, the CN2, generated from pre-immersion of ACFs with Co ions, was characterized by the pyridine-like structures of the six-member rings, quaternary N, and hydrophobicity due to a deficiency of O atoms. On the other hand, CN1 was featured by pyrrolic or amine moieties and pyridine-type N.

**Figure 4:**
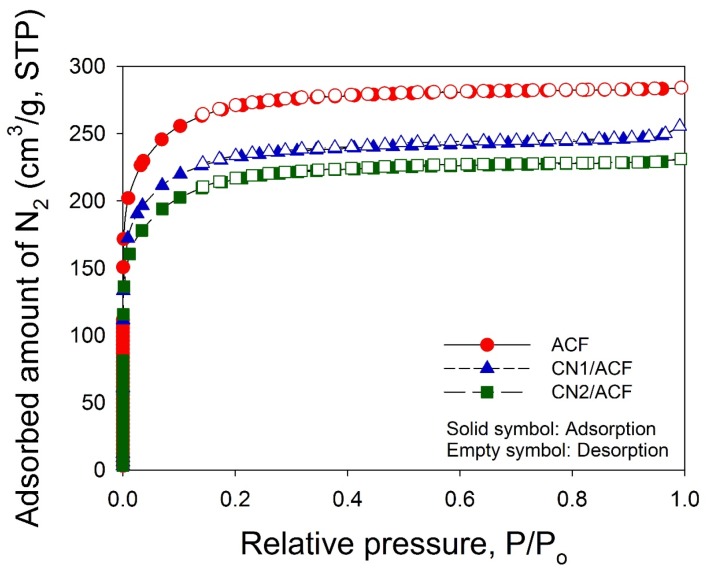
Adsorption isotherms of N_2_ at –196 °C on the samples: (**a**) as-received activated carbon fibers (ACF); (**b**) ACF grafted with N-doped carbon nanotubes (CN1/ACF); (**c**) ACF pre-immersed with cobalt(II) acetate and grafted with N-doped carbon nanotubes (CN2/ACF).

The N_2_ adsorption/desorption isotherms of the samples at –196 °C (Figure 4) indicated that the adsorption isotherms for all samples were essentially type I according to the Brunauer–Emmett–Teller (BET) classification, and the desorption branch almost superimposed on the adsorption branch, indicative of microporosity. The BET specific surface areas of ACF, CN1/ACF, and CN2/ACF were 886, 757, and 709 m^2^/g, respectively (Table 3). The total pore volume and micropore volume for all samples have a similar trend. Although it is believed that grafting CNTs onto the CF surface was an effective method to improve the fiber surface area [16], a contrast was observed in this study. It implied the deposition of catalytic metal particles on ACFs required for CN growth might block the original micropores on the ACFs. Moreover, the effect of the loss of microporosity on the specific surface area and pore volume exceeded the introduction of the defects from CNs.

### 3.2. Adsorption Performance of Various ACF Samples

**Figure 5:**
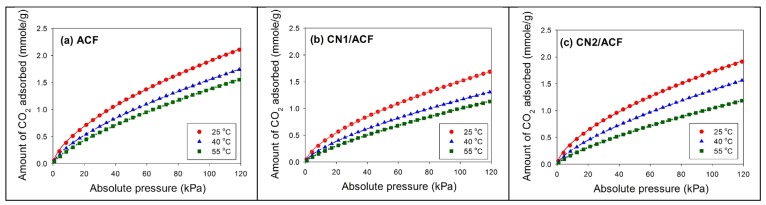
Adsorption isotherms of CO_2_ on the samples at various temperatures: (**a**) as-received activated carbon fibers (ACF); (**b**) ACF grafted with N-doped carbon nanotubes (CN1/ACF); (**c**) ACF pre-immersed with cobalt(II) acetate and grafted with N-doped carbon nanotubes (CN2/ACF). The lines are the fitted curves by the Freundlich equation.

Figure 5 shows the equilibrium data of CO_2_ adsorbed on all samples at different pressures (0–120 kPa) and temperatures (25 °C, 40 °C, 55 °C). The adsorbed amounts increased with increasing pressure, but decreased with increasing temperature, indicating that CO_2_ adsorption was an exothermic reaction. The adsorption amounts of CO_2_ at 25 °C and 1 atm followed the order ACF (1.92 mmol/g) > CN2/ACF (1.75 mmol/g) > CN1/ACF (1.53 mmol/g). Although the adsorption capacity based on the weight of the adsorbent showed the decreased performance after grafting the CNs, the performance based on the specific surface area was different. The adsorption capacities of CO_2_ followed the order CN2/ACF (2.47 μmol/m^2^) > ACF (2.17 μmol/m^2^) > CN1/ACF (2.02 μmol/m^2^) at 25 °C and 1 atm. Comparisons of CO_2_ adsorption in this study with various supporting materials and modifications from the literature are given in Table 4. It was believed that the CNs/ACF could be a promising adsorbent for CO_2_ adsorption after further modification.

**Figure 6:**
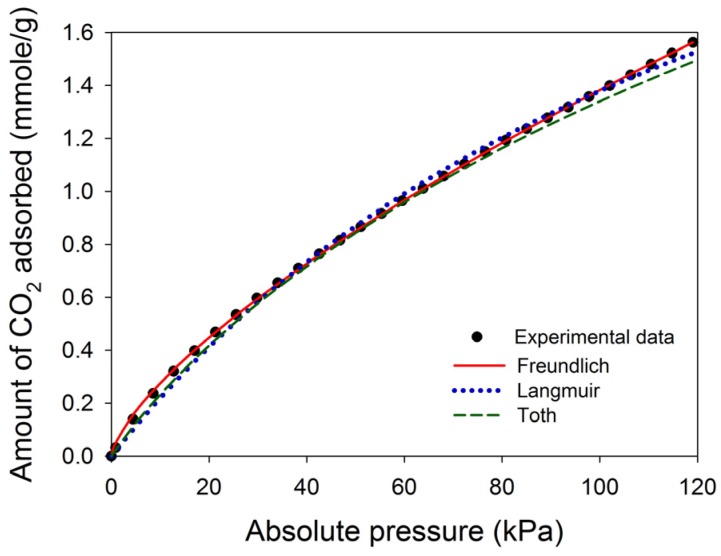
Comparison of the fitted results of three isotherm equations for the adsorption of CO_2_ on CN2/ACF at 40 °C.

The selection of adsorption isotherms has a significant effect on the calculated isosteric enthalpy of adsorption [33]. This study attempted to compare the fitted results of three common adsorption isotherms for the adsorption data, where CN2/ACF was chosen as an example. The results are shown in Table 5. All isotherms could adequately fit the experimental data, but the coefficient of determination (*r*^2^) indicated that the Freundlich equation fit better than the Langmuir and Toth equations at 25–55 °C (*r*^2^ ≥ 0.9997). Figure 6 illustrates the fitted curves for CN2/ACF at 40 °C. Specifically, the fitting of the Langmuir and Toth equations failed at lower and higher pressure ends. Therefore, the Freundlich equation was chosen to fit all adsorption isotherms, and the fitted lines are also shown in Figure 5.

**Figure 7:**
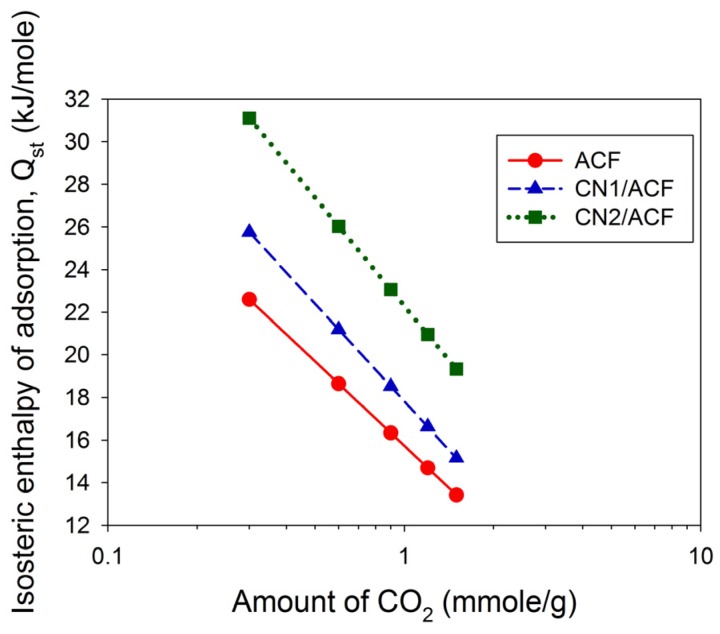
The isosteric enthalpy of the adsorption of CO_2_ on all samples: (**a**) as-received activated carbon fibers (ACF); (**b**) ACF grafted with N-doped carbon nanotubes (CN1/ACF); (**c**) ACF pre-immersed with cobalt(II) acetate and grafted with N-doped carbon nanotubes (CN2/ACF).

The fitted results are summarized in Table 6. The parameter *n* varied between 1.3 and 1.6, indicative of the favorable adsorption. The values of *K*_F_ and *n* decreased with increasing temperatures, consistent with the adsorption capacity of CO_2_. The calculation of the isosteric enthalpy of adsorption (*Q*_st_) follows (shown in Figure 7). The values of *Q*_st_ were less than 40 kJ/mol, implying physical adsorption in the adsorption amounts of 0.3–1.5 mmol/g. The relationship between *Q*_st_ and the amount of CO_2_ adsorbed was linear (Figure 7) if the logarithmic scale of the amount of CO_2_ adsorbed was used. The value of *Q*_st_ followed the order CN2/ACF > CN1/ACF > ACF, implying that the growth of CNs on the ACFs benefit CO_2_ adsorption.

The CO_2_ adsorption capacity and *Q*_st_ were different depending on the sample. The reason could be the residue of the catalysts for the growth of CNs. Since the as-received CN1/ACF and CN2/ACF were used without any purification, the existence of iron or cobalt could block the porosity. Thus, the fact that Lewis-base active sites on the materials increased and the electron-donor properties were enhanced, may have resulted in the increase in the isosteric enthalpy of adsorption. However, the residue of the catalyst was responsible for the lower adsorption capacity.

## 4. Conclusions

The spaghetti-like and randomly oriented N-doped carbon nanotubes (CNs), with high density and homogeneity, were successfully grown onto the polyamide (PAN)-based activated carbon fibers (ACFs). The introduction of CNs improved the smoothness of the surface of ACFs with more anchors. However, this action decreased the specific surface area and pore volume by about 10%–20%, probably due to the deposition of metal catalyst particles on the ACFs required for CN growth, which blocked the original micropores on the ACFs. The growth mechanism of CNs was the tip-growth mode. The pre-immersion of catalytic metal precursors for ACFs did not enhance the degree of graphitization and maintained the oxidation resistance. For CO_2_ adsorption, the specific surface area of the adsorbents may not be the only critical parameter, and the carbonaceous adsorbents with pyridine-like structures of six-member rings were expected to achieve a better capacity [34].

## Figures and Tables

**Table 1:** Surface atomic ratios of the samples from XPS analysis.

Sample	Atomic ratio (%)					N/C	O/C
	C_1s_	N_1s_	O_1s_	Fe_2p_	Co_2p_		
ACF	89.37	2.43	8.20	--	--	0.0272	0.0918
CN1/ACF	88.75	5.66	4.90	0.69	--	0.0638	0.0552
CN2/ACF	93.75	4.13	1.95	0.03	0.14	0.0441	0.0208

**Table 2:** Results of the fits of the XPS N_1s_ region, values given in at% of total intensity.

Binding Energy (eV)	Type	ACF	CN1/ACF	CN2/ACF
395.7	Nitride-like species or aromatic N-imines	--	1.53	2.57
398.4	Pyridine-type N	23.13	23.21	36.29
400.1	Pyrrolic or amine moieties (or pyrrole, pyridone)	17.90	36.73	8.45
401.2	Quaternary N	22.49	8.36	21.20
402.4	Pyridine-N oxides	9.94	9.49	2.45
404.0	Shake-up satellites	2.10	--	6.32
405.0	NO_2_	24.45	20.68	22.72

**Table 3:** Surface characteristics of the samples determined from N_2_ adsorption/desorption isotherms.

Sample	SSA (m^2^/g)	*S*_mi_^α^ (m^2^/g)	*S*_ext_^β^ (m^2^/g)	*V*_t_^γ^ (cm^3^/g)	*V*_mi_^η^ (cm^3^/g)	*V*_me_^ϕ^ (cm^3^/g)	*V*_ma_^ξ^ (cm^3^/g)	Mean Pore Size ^ζ^ (nm)
ACF	886	639	247	0.4395	0.3076	0.0758	0.0561	1.984
CN1/ACF	757	547	210	0.3951	0.2652	0.0748	0.0551	2.087
CN2/ACF	709	478	231	0.3577	0.2312	0.0722	0.0543	2.019

^α^ *S*_mi_ was determined by *t*-plot method. ^β^ *S*_ext_ was obtained by subtracting *S*_mi_ from SSA. ^γ^ *V*_t_ represents the single point total pore volume at *P*/*P*_o_ ≈ 0.99. ^η^ *V*_mi_ was determined by t-plot method. ^ϕ^ *V*_me_ was calculated by BJH method. ^ξ^ *V*_ma_ was found by subtracting *V*_me_ and *V*_mi_ from *V*_t_. ^ζ^ Mean pore size was obtained by 4*V*_t_/SSA.

**Table 4:** Comparisons of CO_2_ adsorption in the present study with various supporting materials and modifications from the literature.

Supporting Materials	Amine Type	Temp. (°C)	Concentration of CO_2_	CO_2_ Adsorption (mmol/g)	Source
Granular activated carbons	NH_3_ (with pre-oxidation)	30	1 atm	~1.50	[28]
Silica-coated multi-walled CNTs (MWCNTs)	Polyethyleneimine	25	1 bar	1.41	[29]
Fluorinated graphene	Ethylenediamine	0	1.1 bar	1.16	[30]
MWCNTs	3-Aminopropyl-triethoxysilane	20	15%	0.98	[12]
Granular activated carbons	NH_3_	30	1 atm	~1.7	[31]
Carbon monolith	Lysine	25	1 atm	3.13	[32]
ACFs	—	25	1 atm	1.92	This study
ACFs	CN1	25	1 atm	1.53	This study
ACFs	CN2	25	1 atm	1.75	This study

**Table 5:** Fitted parameters of three isotherm equations for CO_2_ adsorption on CN2/ACF.

Temp. (^o^C)	Freundlich			Langmuir			Toth			
	*K*_F_	*n*	*r*^2^	*q*_m_	*K*_L_	r^2^	*q*_T_	*K*_T_	*t*	*r*^2^
25	0.0987	1.61	0.9997	3.39	0.0103	0.9957	9.86	0.0077	0.43	0.9982
40	0.0550	1.43	0.9999	3.34	0.0070	0.9973	6.30	0.0046	0.62	0.9954
55	0.0336	1.34	0.9999	2.95	0.0054	0.9985	3.76	0.0045	0.82	0.9968

**Table 6:** Fitted parameters in the Freundlich equation for CO_2_ adsorption at different temperatures.

Sample	Temperature (^o^C)	*K*_F_ (mmol/g/kPa^1/n^)	*n* (-)	*r*^2^ (-)
ACF	25	0.1051	1.59	0.9998
	40	0.0689	1.48	0.9998
	55	0.0490	1.38	0.9998
CN1/ACF	25	0.0813	1.58	0.9998
	40	0.0492	1.46	0.9998
	55	0.0315	1.33	0.9999
CN2/ACF	25	0.0987	1.61	0.9997
	40	0.0550	1.43	0.9999
	55	0.0336	1.34	0.9999
